# *Minos* and *Restless* transposon insertion mutagenesis of psychrotrophic fungus for red pigment synthesis adaptive to normal temperature

**DOI:** 10.1186/s40643-022-00604-5

**Published:** 2022-11-04

**Authors:** Fengning Lu, Yanna Ren, Lulu Ding, Jian Lu, Xiangshan Zhou, Haifeng Liu, Nengfei Wang, Menghao Cai

**Affiliations:** 1 State Key Laboratory of Bioreactor Engineering, East China University of Science and Technology, Shanghai, 200237 China; 2 China Resources Biopharmaceutical Co., Ltd, Unit 601, Building No. 2, YESUN Intelligent Community III, Guanlan Street, Shenzhen, China; 3 China Resources Angde Biotech Pharma Co., Ltd, 78 E-Jiao Street, Liaocheng, 252201 Shandong China; 4 First Institute of Oceanography, Ministry of Natural Resources, Qingdao, 266061 China; 5 Shanghai Frontiers Science Center of Optogenetic Techniques for Cell Metabolism, East China University of Science and Technology, 130 Meilong Road, Shanghai, 200237 China

**Keywords:** Polar fungi, Psychrotroph, *Geomyces* sp., Red pigment, Transposon mutagenesis

## Abstract

The polar psychrotrophic fungus *Geomyces* sp. WNF-15A can produce high-quality natural red pigment for the potential use as edible pigment. However, it shows low-temperature-dependent synthesis of red pigment, which limits its large-scale industrial applications due to the difficult and high-cost bioprocess control. This study aims to develop transposon-mediated mutagenesis methods to generate mutants that are able to synthesize red pigment at normal temperature. Four transposable systems, including single and dual transposable systems, were established in this fungus based on the *Minos* from *Drosophila hydei* and the *Restless* from *Tolypocladium inflatum*. A total of 23 production-dominant mutants and 12 growth-dominant mutants were thus obtained by constructed transposable systems. At 14 °C and 20 °C, the MPS1 mutant strain achieved the highest level of red pigment (OD_520_ of 43.3 and 29.7, respectively), which was increased by 78.4% and 128.7% compared to the wild-type, respectively. Of note, 4 mutants (MPS1, MPS3, MPS4 and MPD1) successfully synthesized red pigment (OD_520_ of 5.0, 5.3, 4.7 and 4.9, respectively) at 25 °C, which broke the limit of the wild-type production under normal temperature. Generally, the dual transposable systems of *Minos* and *Restless* were more efficient than their single transposable systems for mutagenesis in this fungus. However, the positive mutation ratios were similar between the dual and single transposable systems for either *Minos* or *Restless*. This study provides alternative tools for genetic mutagenesis breeding of fungi from extreme environments.

**Graphical Abstract:**

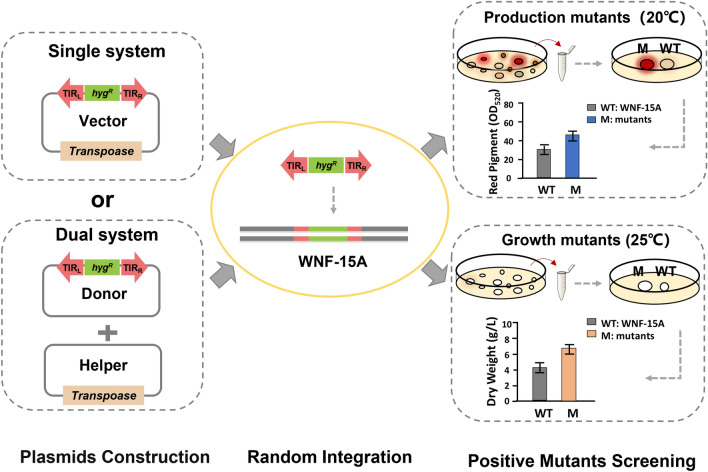

**Supplementary Information:**

The online version contains supplementary material available at 10.1186/s40643-022-00604-5.

## Introduction

Polar regions, including the Antarctic, the Arctic and its surroundings, are among the coldest habitats on Earth. Apart from extreme cold, the organisms that survive in polar regions have to adapt to desiccation, strong winds, low water availability, high UV radiation, multiple freeze–thaw cycles, etc. (Hayward et al. 2021). Biodiversity recognized from the polar regions is still limited because of the extremely harsh conditions. However, some psychrophilic and psychrotrophic microorganisms can inhabit in the polar regions with special adaptation mechanisms, such as the secretion of antifreeze proteins, fatty acids, polyols, pigment, exopolysaccharides, cold-active enzymes, novel bioactive compounds, etc. (Rosa et al. 2019; Tsuji et al. 2013; Wong et al, 2019; Zucconi et al. 2020). Recently, studies have also focused on bioactive extracts from polar fungi, which can effectively inhibit pathogenic bacteria, viruses, parasites, tumors, etc. (Dos et al. 2020; Furbino et al. 2014; Gonçalves et al. 2015; Purić et al. 2018; Vieira et al. 2018; Wu et al. 2013). Nevertheless, commercial products generated from polar microorganisms are still limited. The special physiological characteristics of the polar microorganisms bring many difficulties in laboratory- and industrial-scale culture.

*Geomyces* sp. WNF-15A is a polar fungus isolated from Antarctic soil. It can produce an extracellular red pigment with a major content of geomycamine at low temperature (Wang et al. 2013). The red pigment is of good water solubility and owns higher extinction coefficient compared to the commercially high-end cochineal red pigment (Jin et al. 2014). Importantly, this Antarctic *Geomyces* red pigment shows better stability than cochineal red pigment when used in reducing agents, foods, acid and alkali environments. Also, it has good tolerance to oxidants, ultraviolet rays and various metal ions (Jin et al. 2014). Moreover, median lethal dose (LD50) of the Antarctic *Geomyces* red pigment reached over 15 000 mg/kg and thus was identified as nontoxic in China (Wang et al. 2013). And there was no harmful byproduct detected from *Geomyces* sp. WNF-15A culture compared with the widely used *Monascus* red pigment in Asia (Ding et al. 2021). Therefore, the Antarctic *Geomyces* red pigment has a great potential for industrial use. Nevertheless, *Geomyces* sp. WNF-15A is a typical polar psychrotrophic fungus. It was apt to produce the red pigment at temperature lower than 14 °C, but its synthesis was severely impaired at above 20 °C (even absolutely blocked at 25 °C) (Huang et al. 2020; Ding et al. 2021). This cold-adaptive production trait brings difficult and high-cost bioprocess control in large-scale fermentation.

To obtain microorganisms adapting for industrial use, random mutagenesis strategies have been commonly used to improve mutation rates and screen beneficial varieties (Kodym and Afza, 2003). Although random mutagenesis by chemical and physical methods is easy-to-use, it is generally hard to track the mutation sites in microbial genomes. Transposon insertion mutation represents an alternative mutation method for gene inactivation and phenotype change, which is user-friendly, economically competitive, traceable, and almost no preferences for any special locus, thus aiding in screening, analyzing and identifying gene functions in species with unclear genetic background (Ding et al. 2021; Kim and Pyykko 2011; Paun and Kempken 2015; Sandoval-Villegas et al. 2021). Unlike the wide use in bacteria, identification and application of transposons in fungi are limited. Till now, only a few transposable elements (TEs) were used to filamentous fungi, mostly the *Tc1*/*mariner* and *hAT* superfamily members. For instance, the *Tc1*/*mariner* superfamily TEs of *Fot1* and *impala* from *Fusarium oxysporum* functioned normally in *Aspergillus nidulans* (Nicosia et al. 2001). The *impala* TE was also active in other hosts including *Aspergillus fumigatus*, *Penicillium griseoroseum*, and *Magnaporthe grisea* (Carr et al. 2010; de Queiroz and Daboussi 2003; Villalba et al. 2001), despite that it tended to insert into the unessential regulatory or noncoding regions (Deschamps et al. 1999).

Recently, a *Minos*-based transposable system with the transposition frequency closed to the *impala* was developed (Evangelinos et al. 2015). The *Minos*, a DNA transposon belonging to *Tc1*/*mariner* superfamily, was isolated from the genome of *Drosophila hydei* and widely active in this species (Metaxakis et al. 2005). It is approximately 1775-bp in full-length, carrying a 60-bp long intron and two flanking 255-bp inverted terminal repeats (TIRs) (Franz and Savakis 1991). The insertion sites of *Minos* are fully random in host genome wide despite of a slight TA dinucleotides preference (Bellen et al. 2011). Similar to other transposons, it leaves a short footprint (6-bp) at the excision site (Pavlopoulos et al. 2007). Indeed, the activity of *Minos* is not limited to insect species, it also has been shown to be active in other species, ranging from chordate *Ciona intestinalis* (Sasakura et al. 2003), human cells (Klinakis et al. 2000; Zagoraiou et al. 2001), mouse cells (Drabek et al. 2003) and even fungi like *Aspergillus nidulans* (Evangelinos et al. 2015). The transposition frequency of the *Minos* element was comparable to *impala*, an efficient and common tool for gene functional analysis in fungi (Evangelinos et al. 2015). Besides, the first fungal *hAT* transposon *Restless* found in *Tolypocladium inflatum* is 4097-bp in length and harbors two TIRs of about 20-bp (Kempken and Kück 1998, 2000). It can produce an 8-bp target site duplication at the integration site after working in hosts. Similarly, the *Restless*-based system has been confirmed to maintain the activity of excision and integration in foreign hosts containing *Neurospora crassa* and *Penicillium chrysogenum* (Paun and Kempken 2015; Windhofer et al. 2000, 2002). Both *Minos* and *Restless* belong to “cut–paste” DNA transposons, which enable a known cassette or sequence to be removed from donor location and insert randomly at a new locus in target genome.

This study aims to develop *Minos*- and *Restless*-based transposon insertion mutation methods in the *Geomyces* sp. WNF-15A. By this way, we attempted to obtain mutants with improved red pigment production and normal temperature adaptation derived from this polar fungus. With the visible colony growth and color appearance, mutants of high-production or normal-temperature adaptation can be easily identified in solid agar plate followed by liquid culture verification. This work provides an alternative method for strain improvement of *Geomyces* sp. WNF-15A and other psychrotrophs or psychrophiles from extreme environments.

## Materials and methods

### Strains and culture conditions

The wild-type *Geomyces* sp. WNF-15A strain was provided by Dr. Nengfei Wang, First Institute of Oceanography, Ministry of Natural Resources, China. The wild-type and mutant strains were stored in 20% (w/v) glycerol at − 80 °C in our laboratory. *Escherichia coli* Top 10 was purchased from Invitrogen and preserved in our laboratory. The Luria–Bertani [10 g/L NaCl, 10 g/L tryptone, 5 g/L yeast extract and 20 g/L agar (solid media)] was used to culture *E. coli*. The seed medium (10 g/L glucose, 20 g/L maltose, 20 g/L mannitol, 10 g/L sodium glutamate, 6 g/L yeast extract paste, 0.3 g/L MgSO_4_·7H_2_O, and 0.5 g/L KH_2_PO_4_) was used to culture *Geomyces* sp. WNF-15A and its mutants, and 30 g/L agar was added for normal solid media. While 20 g/L and 7.5 g/L agar was added for mutants screening. For the fermentation production of red pigment, the wild-type and mutants were cultured in medium S (28 g/L soluble starch and 1.85 g/L tryptone).

To culture fungal strains, 100 μL spore suspension was inoculated into 5 mL seed medium and cultured in the dark at 20 °C and 130 rpm for 36 h. Then 1 mL inoculum was cultured in 250-mL shake flasks containing 50 mL seed medium and cultured for 3 days under the same conditions to prepare the first order seed. For this process, 20 glass beads with a diameter of 4 mm were added to disperse the mycelia. The obtained broth was transferred to 250-mL shake flasks containing 50 mL seed medium at 10% (v/v) inoculation and cultured under the same conditions for 36 h to prepare the second-order seed. Then 200 μL culture broth was evenly spread on agar medium and cultured in fungal incubator with 80% humidity at 20 °C or 25 °C. For shake flask fermentation, the second-order seed was inoculated in 250-mL shake flasks containing 50 mL medium S by 6% (v/v), and 20 glass beads with a diameter of 4 mm were added. It was then cultured in the dark at 130 rpm for the desired days (Huang et al. 2020). Three biological replicates were arranged for each experiment. Besides, *E. coli* Top 10 strain was incubated in Luria–Bertani (LB) medium at 180 rpm and 37 °C.

### Construction of plasmids

We firstly removed the open reading frame of *CAS9* from the plasmid pFC332 (Nødvig et al. 2015) to generate a backbone vector pFC000. The pFC332 was donated by Prof. Reinhard Fischer of the Karlsruhe Institute of Technology, Germany. The synthesized coding sequence of *Minos* transposase (GenBank: Z29102.1) was inserted into the pFC000 plasmid digested with *Pac*I and *Sph*I by GenScript (Nanjing) Co., Ltd. Two synthesized TIRs of *Minos* were fused at the 5′ flanking site of the *TrpC* promoter and the 5′ flanking site of the *TrpC* terminator by seamless cloning after double restriction enzyme digestion with *BamH*I and *Nar*I. By this way, the plasmid pFC000-M was obtained. The construction of plasmid pFC000-R was the same as the plasmid pFC000-M, except that the transposase (intron not removed from its coding gene) and TIRs were from *Restless* (GenBank: Z69893.1). Expression cassette of hygromycin resistance gene (*hyg*^*R*^) was amplified by primers of HM-F1/HM-R1 and inserted into the pFC000-M plasmid digested with *Nco*I to obtain the plasmid of pFC000-MH. Similarly, *hyg*^*R*^ was amplified by primers of HR-F1/HR-R1 and inserted into the pFC000-R plasmid digested with *Stu*I to obtain the plasmid of pFC000-RH.

Dual transposable systems were also constructed, for which the donor plasmid carries TIRs and the helper plasmid carries the transposase gene. The pFC000-MH was digested with *BamH*I and *Pac*I to remove transposase cassette of *Minos*, and a small noncoding fragment amplified by primers of HM-DF1/HM-DR1 was inserted, generating a donor plasmid of pFC-MD for *Minos* transposable system. The pFC000-M was digested with *Age*I and *Pas*I to remove the TIRs and the sequence between them, and a small noncoding fragment amplified by primers of HM-HF1/HM-HR1 was inserted, generating a helper plasmid of pFC-MH for *Minos* transposable system. The pFC000-R plasmid was digested with *Sph*I and *Pac*I, and a small noncoding fragment amplified by primers of HR-DF1/HR-DR1 was inserted, generating a mid-donor plasmid of pFC-RD1. Then the pFC-RD1 plasmid was digested with *Stu*I, and the *hyg*^*R*^ cassette amplified by primers of HR-F1/HR-R1 was introduced to obtain the donor plasmid of pFC-RD2 for *Restless* transposable system. The pFC000-R plasmid was digested with *Age*I and *Pas*I to remove the TIRs and the sequence between them, and a small noncoding fragment amplified by primers of HR-HF1/HR-HR1 was inserted, generating a helper plasmid of pFC-RH for *Restless* transposable system. Primers used for the construction of plasmids are listed in Additional file 1: Table S1. The constructed plasmids were amplified and purified from *E. coli* Top 10. Transformation and other standard DNA operations followed protocols described previously (Green et al. 2012).

### Fungal transformation

Protoplasts preparation and PEG-mediated transformation of *Geomyces* sp. WNF-15A were performed as described previously (Ding et al. 2021). The transposable plasmid was introduced into protoplasts of the wild-type strain, and the protoplasts were regenerated by incubation at solid seed medium plate with 20 g/L agar in the dark and, at 80% humidity and 20 °C or 25 °C for 36–48 h. Then the upper layer medium was covered using solid seed medium plate with 7.5 g/L agar and 30 μg/mL hygromycin B under the same conditions for 5–7 days.

### Molecular identification of mutants

Positive transformants were firstly screened by hygromycin B. Then transformants losing plasmids were identified by spore PCR with primer pairs of Am-F/Am-R. Genomic DNA was extracted using the Plant Genomic DNA Extraction Kit (Tiangen). The spore PCR were performed as described previously (Ding et al. 2021). Transformants without carrying plasmids were further used for genotypic verification. Primers used for PCR analysis are listed in Additional file 1: Table S1. The theoretical PCR products were 1208 bp (the insertional fragment for pFC000-MH using primers of Mi-F/Mi-R), 1153 bp (the insertional fragment for pFC000-RH using primers of Re-F/Re-R), 1553 bp (the insertional fragment for pFC-MD using primers of MD-F/MD-R), and 1153 bp (the insertional fragment for pFC-RD2 using primers of RD-F/RD-R), respectively.

### Data analyses

The red pigment was quantified by monitoring absorbance at 520 nm (OD_520_) using a spectrophotometer. The 1 mL culture broth was taken periodically and centrifuged at 12,000 rpm for 3 min. Then the absorbance value of the diluted supernatant was measured at OD_520_. And the growth of strains was measured by biomass dry weight as described previously (Huang et al. 2020). All trials were conducted in triplicate. Statistical significance was calculated by *p*-values using one-way analysis of variance (ANOVA), and results with *p* < 0.05 were deemed significant.

## Results and discussion

### Construction of single and dual transposable systems of *Minos* and *Restless* for *Geomyces* sp. WNF-15A

*Minos* and *Restless* have been shown to be able to transpose in heterologous fungi and have the potential to work in the polar fungus *Geomyces* sp. WNF-15A. We therefore developed transposable systems based on *Minos* and *Restless* elements for *Geomyces* sp. WNF-15A, including single and dual transposable systems for either transposon.

**Fig. 1:**
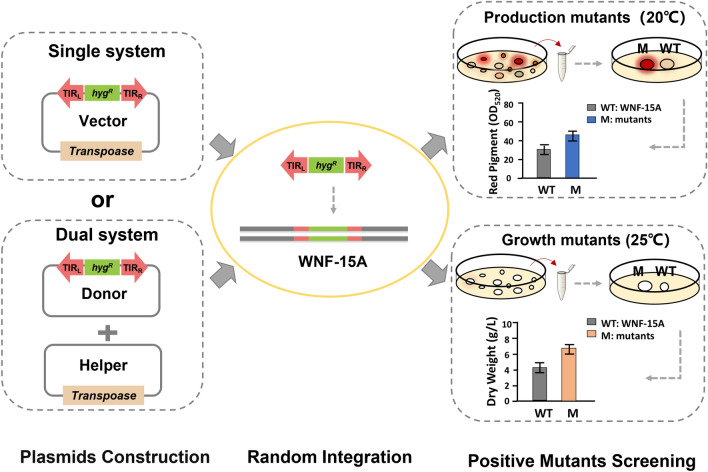
Strategy for the construction of transposable systems and screening of the positive mutants. Single system owns a single plasmid carrying transposase gene cassette and a known fragment with hygromycin resistance gene (*hyg*^*R*^) and flanked by left (*TIR*_*L*_) and right (*TIR*_*R*_) terminal inverted repeats of transposons. Dual system consists of a donor plasmid with insertion fragment and a helper plasmid with transposase gene cassette. The transposase achieves transposition by excising the “known fragment” from the original plasmid and randomly integrating a new locus in host genome to select progeny changed in phenotype

The general construction idea was to introduce a selection marker of *hyg*^*R*^ between the flanking TIRs by in-fusion cloning, with the purpose of improving the screening efficiency. The single transposable system depends on an episomal plasmid, which mainly carries the transposase-encoding gene and *hyg*^*R*^ flanked by TIRs (Fig. 1). Considering that a large-size plasmid may not easily enter into the cell, which in turn affects the mutation effect, the dual transposable system was further constructed. Unlike the single transposable system, the dual system consists of a donor episomal plasmid carrying *hyg*^*R*^ flanked by TIRs and a helper episomal plasmid carrying a transposase-encoding gene (Fig. 1).

Similar to most DNA transposons, *Minos* and *Restless* move into target genome by "cut–paste" mechanism (Pavlopoulos et al. 2007; Munoz-Lopez and Garcia-Perez 2010). After introducing the single or dual transposable plasmids into the host, the expressed transposase will excise the intact *TIR*_*L*_-*hyg*^*R*^-*TIR*_*R*_ fragment and insert into the host genome. In turn, changes in the host phenotype may lead to the target mutants of interest. Generally, these episomal plasmids will easily vanish during antibiotic-free culture and thus the mutated genotype and phenotype can be stably inherited. The color of positive mutants in red pigment were redder than wild-type on the plate, and the diameter of positive mutants in growth were bigger than wild-type on the plate (Fig. 1). The phenotype of screening mutants based on plate culture was further verified in liquid fermentation.

### Analysis of positive mutants obtained by the single transposable systems of *Minos* and *Restless*

The transposon transformation was conducted at 20 °C and 25 °C to obtain the mutants producing red pigment adapted to normal temperature. With these constructed *Minos* and *Restless* transposable systems, a series of transposon insertion mutants can be screened. Among them, 134 mutants and 6 mutants were screened by single transposable systems at 20 °C and 25 °C, respectively (Table 1). We mainly care about the positive mutants with red pigment production and colony growth at different temperatures. The red pigment mutants were screened by directly observing colony color on the agar plate, for which mutant colonies redder than the wild-type were collected. Similarly, the growth mutants were screened by measuring colony diameter on the agar plate, for which mutant colonies bigger than the wild-type were collected.

**Table 1:** The efficiency of *Minos* and *Restless* transposons in *Geomyces* sp. WNF-15A

Transposable systems	*Minos*		*Restless*	
	Single	Dual	Single	Dual
The number of transformants	1041	830	339	579
The number of transposable mutants	101	99	39	76
The number of transposable mutants screened at 20 °C	97	95	37	72
The number of transposable mutants screened at 25 °C	4	4	2	4
The efficiency of transposition (%)	9.7	11.9	11.5	13.1
The number of positive mutants in red pigment^a^	7	6	3	7
The number of positive mutants in growth^b^	5	5	1	1
The positive mutation ratio (%)	11.9	11.1	10.3	10.5

^a^The mutants with variation percentage over 10% in red pigment production were considered as positive mutants, including MPS1–MPS10 and MPD1–MPD13

^b^The mutants with variation percentage over 15% in cell growth were considered as positive mutants, including MPS1, MPS3, MPS4, MGS1, MGS3, MGS6, MPD1, MPD2, MPD4, MGD2, MGD4, and MGD6

**Fig. 2:**
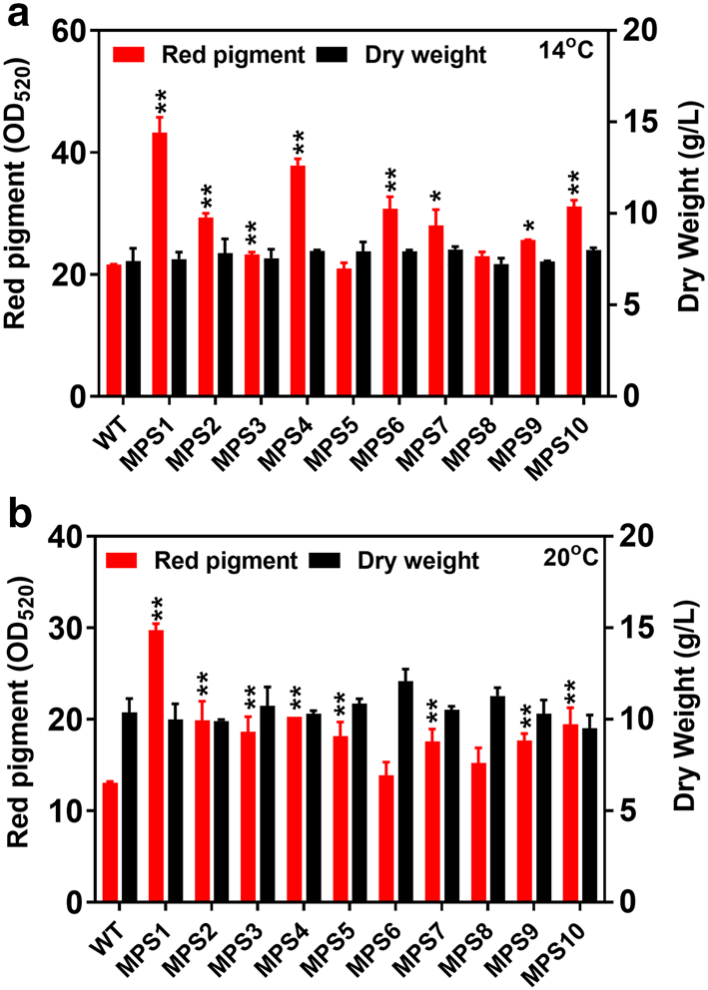
Red pigment production and cell growth of MPS1–MPS10 mutants obtained by single transposable systems. **a** The red pigment production and its dry weight of the mutants at 14 °C. **b** The red pigment production and its dry weight the mutants at 20 °C. The value of the bar represents the highest pigment level or the highest biomass dry weigh at culture duration. Determination of absorbance value of red pigment at wavelength of 520 nm (OD_520_). Data of significance were shown in **P < 0.01 and *P < 0.05

As a result, 13 transposable positive mutants were obtained by single transposable systems of *Minos* and *Restless*. Therein, the advantaged mutants in red pigment, MPS1–MPS10, were screened from 20 °C culture (Additional file 1: Fig. S1a), and the dominant mutants in colony growth, MGS1, MGS3 and MGS6, were screened from 25 °C culture (Additional file 1: Fig. S1b). Afterwards, the MPS1–MPS10 mutants were cultured in flasks for production of red pigment under different temperatures of 14 °C, 20 °C and 25 °C. The results indicated that the red pigment production of most mutants, i.e., MPS1, MPS2, MPS3, MPS4, MPS6, MPS7, MPS9 and MPS10, were enhanced compared with the wild-type at 14 °C (Additional file 1: Fig. S2a). Among them, the red pigment of MPS1 achieved the highest level (OD_520_ of 43.3) at 14 °C, which was 78.4% higher than that of wild-type (Fig. 2a), while the cell growth of various mutants showed on obvious difference from the wild-type (Fig. 2b). When cultured at 20℃, the red pigment production of MPS1–MPS10 decreased by 14–55% compared to their productions at 14 °C (Additional file 1: Fig. S2b). However, the maximum biomass dry weight of these mutants increased by 19–56% at 20 °C compared to their biomass at 14℃ (Fig. 2 and Additional file 1: Fig. S2). The results indicated that the red pigment production of most mutants, i.e., MPS1, MPS2, MPS3, MPS4, MPS5, MPS7, MPS9 and MPS10, were enhanced compared with the wild-type at 20 °C (Additional file 1: Fig. S2b). The MPS1 mutant still achieved the highest level of red pigment (OD_520_ of 29.7) at 20 °C, which was 128.7% higher than the wild-type.

**Fig. 3:**
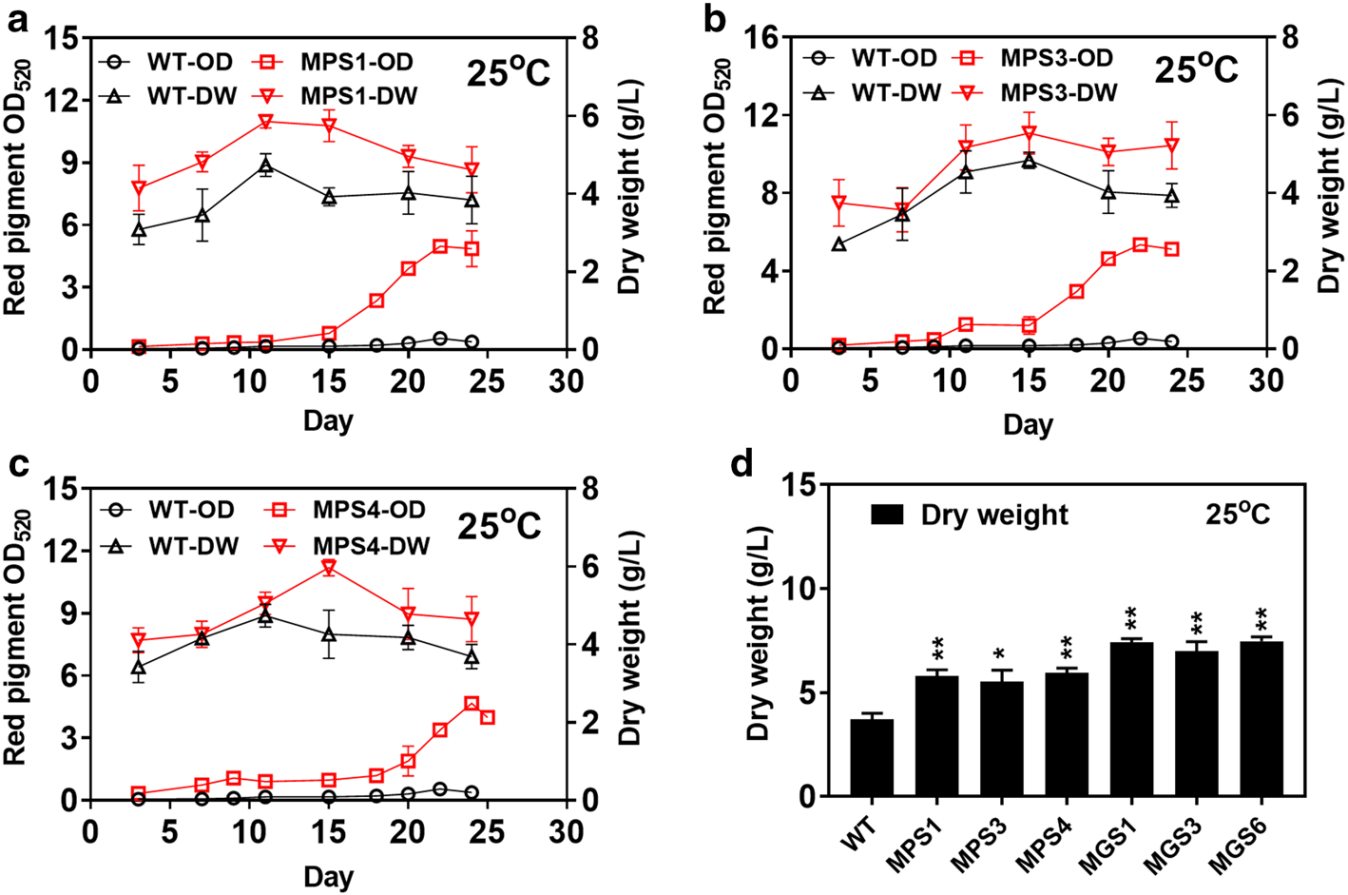
Red pigment production and cell growth of mutants obtained by single transposable systems at 25 °C. The red pigment production of MPS1 (**a**), MPS3 (**b**) and MPS4 (**c**) were shown. **d** The dry weight from mutants of MPS1, MPS3, MPS4, MGS1, MGS3, MGS6. The value of the bar represents the highest pigment level or the highest biomass dry weigh at culture duration. WT, wild-type; DW, biomass dry weight. Determination of absorbance value of red pigment at wavelength of 520 nm (OD_520_). Data of significance were shown in **P < 0.01 and *P < 0.05

At the normal temperature of 25 °C, most of the above mutants were still unable to synthesize red pigment, except for MPS1, MPS3 and MPS4 (OD_520_ of 5.0, 5.3 and 4.7, respectively) (Fig. 3a–c). These mutants overcame the natural repression of red pigment synthesis when temperature was over 25 °C. And the cell growth of these three mutants was also better than the wild-type at 25 °C. It proved the effectiveness of the engineered *Minos* and *Restless* transposable systems despite of the low red pigment production of the mutants at 25 °C. On the other hand, the maximum diameter of MGS1, MGS3 and MGS6 colonies maintained around 10–13 mm with significant growth improvement (*p* < 0.01) comparing with that of the wild-type (9–11 mm). Further, biomass dry weight of MGS1, MGS3 and MGS6 reached around 6–8 g/L, which was also higher than the wild-type (Additional file 1: Fig. S3). Among them, the MGS6 mutant achieved a maximum biomass dry weight of 7.5 g/L at 25 °C, which was 45.1% higher than the wild-type (Fig. 3d). Of note, the obtained biomass of MGS6 at 25 °C even reached an equivalent level of the wild-type at 14 °C. Generally, the mutants of MPS1–MPS10 with variation percentage over 10% in red pigment production were regarded as effective mutants (Additional file 1: Fig. S4a and c). The mutants of MPS1, MPS3, MPS4, MGS1, MGS3 and MGS6 with variation percentage over 15% in cell growth were recorded as positive mutants (Additional file 1: Fig. S4b, d and e).

### Analysis of positive mutants obtained by the dual transposable systems of *Minos* and *Restless*

**Fig. 4:**
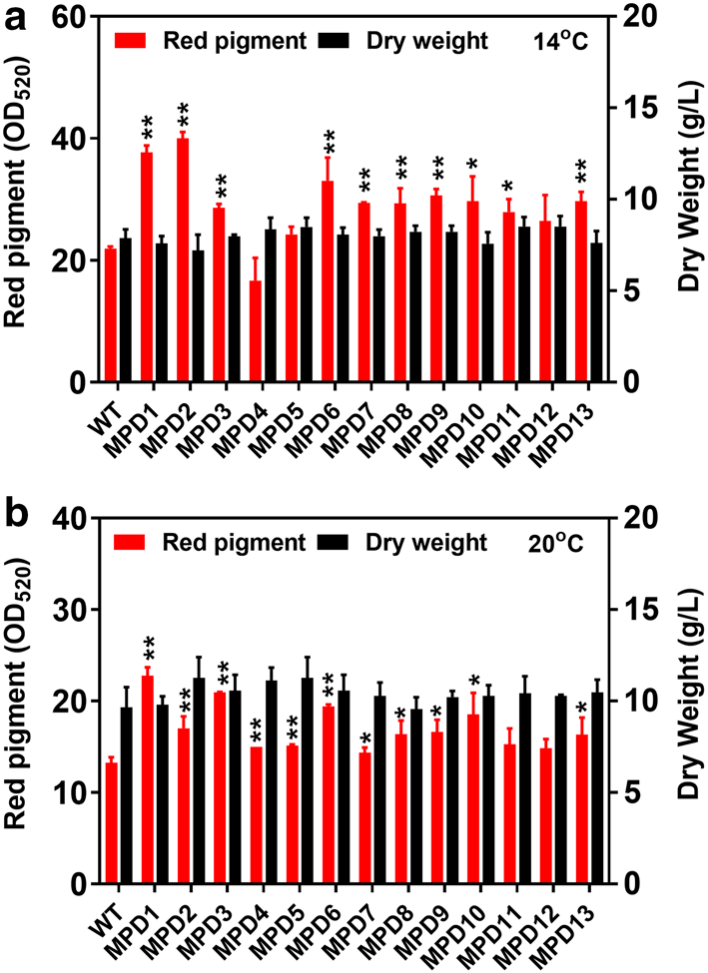
Red pigment production and cell growth of MPD1–MPD13 mutants obtained by dual transposable systems. **a** The red pigment production and its dry weight of the mutants at 14 °C. **b** The red pigment production and its dry weight the mutants at 20 °C. The value of the bar represents the highest pigment level or the highest biomass dry weigh at culture duration. Determination of absorbance value of red pigment at wavelength of 520 nm (OD_520_). Data of significance were shown in **P < 0.01 and *P < 0.05

With the dual transposable systems, full transposable elements were divided into two parts and constructed into the helper and donor plasmids. Therefore, the size of individual plasmid was reduced and then co-transformed into the fungal cells. The 167 mutants and 8 mutants were screened by the dual transposable systems of *Minos* and *Restless* at 20 °C and 25 °C, respectively (Table 1). Among them, a total of 16 positive mutants were obtained. These mutants contained the red pigment dominant MPD1–MPD13 screened from 20 °C culture (Additional file 1: Fig. S5a) and the growth-dominant MGD2, MGD4 and MGD6 screened from 25 °C culture (Additional file 1: Fig. S5b). The mutants of MPD1–MPD13 were then cultured for production of red pigment under different temperatures of 14 °C, 20 °C and 25 °C. At 14 °C, all mutants but not MPD4 produced higher titres of red pigment than the wild-type (Additional file 1: Fig. S6a). Among them, the red pigment of MPD2 achieved the highest level (OD_520_ of 40.0) at 14 °C, which was 82.9% higher than that of the wild-type (Fig. 4a) and a bit lower than that of MPS1 (OD_520_ of 43.3) obtained by the single transposable system (Additional file 1: Fig. S2a), while the cell growth of various mutants showed on obvious difference from the wild-type (Fig. 4b). When cultured at 20℃, the red pigment production of MPD1–MPD13 decreased by 10–68% compared to their productions at 14 °C (Additional file 1: Fig. S6b). However, the maximum biomass dry weight of these mutants increased by 12–53% at 20 °C compared to their biomass at 14 °C. The MPD1 mutant achieved the highest level of red pigment (OD_520_ of 22.8) at 20 °C, which was 82.9% higher than the wild-type (Fig. 4b), while the cell growth of various mutants also showed on obvious difference from the wild-type but not MPD2 and MPD4 (Fig. 4b).

**Fig. 5:**
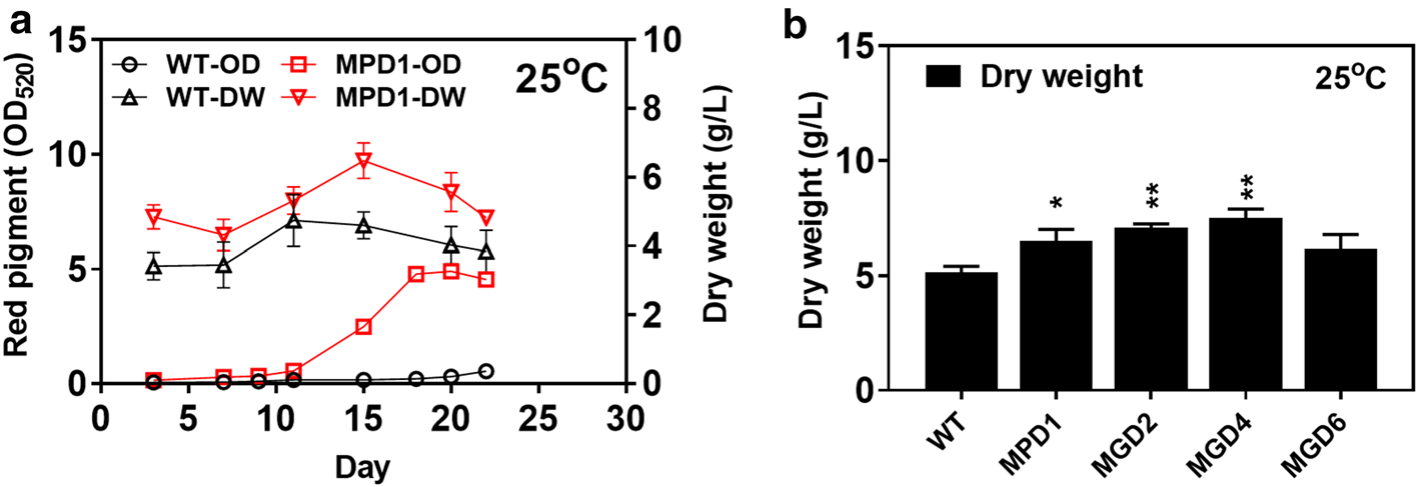
Red pigment production and cell growth of mutants obtained by dual transposable systems at 25 °C. **a** The red pigment production of MPD1. **b** The dry weight of MPD1, MGD2, MGD4, MDS6. The value of the bar represents the highest biomass dry weigh at culture duration. WT, wild-type; DW, biomass dry weight. Determination of absorbance value of red pigment at wavelength of 520 nm (OD_520_). Data of significance were shown in **P < 0.01 and *P < 0.05

For the dual transposable systems, only MPD1 was able to produce red pigment (OD_520_ of 4.9) at 25 °C, with production level close to the MPS1, MPS3 and MPS4 (OD_520_ of 5.0, 5.3 and 4.7, respectively) from the single transposable systems (Fig. 5a). And the cell growth of MPD1 also better than the wild-type at 25 °C (Fig. 5a). In addition, the maximum diameter of MGD2, MGD4 and MGD6 colonies reached around 10–12 mm with significant growth improvement (*p* < 0.01) comparing with that of the wild-type (8–10 mm) (Additional file 1: Fig. S5b). Biomass dry weight of MGD2, MGD4 and MGD6 were also analyzed in culture at 25 °C (Additional file 1: Fig. S7). The MGD4 mutant achieved a maximum biomass dry weight of 7.5 g/L at 25 °C, which was 46.7% higher than the wild-type (Fig. 5b) and similar to the MGS6 mutant obtained from the single transposable system. On the whole, the mutants of MPD1–MPD13 with red pigment production change greater than 10% were considered as positive mutants (Additional file 1: Fig. S8a, c). The mutants of MPD1, MPD2, MPD4, MGD2, MGD4 and MGD6 with cell growth change greater than 15% were recorded as positive mutants (Additional file 1: Fig. S8b, d and e).

### Efficiency analysis of the single and dual transposable systems of *Minos* and *Restless*

The efficiency of the single and dual transposable systems of *Minos* and *Restless* in *Geomyces* sp. WNF-15A was analyzed and summarized from a total of 1871 *Minos* transformants and 918 *Restless* transformants (Table 1). Generally, the number of transformants and the efficiency of transposition from the dual transposable systems were superior than the single transposable systems, especially for the *Restless* transposon. The number of transformants of *Restless* in the dual transposable system was about twofold higher than its single transposable system. The transposition efficiency of *Minos* and *Restless* dual transposable systems reached 11.9% and 13.1%, which were higher than their corresponding single transposable systems, respectively. However, for each transposon, the positive mutation rate was similar between the single transposable system and the dual transposable system, i.e., ~ 11% for the positive mutation rate of *Minos* and ~ 10% for the positive mutation rate of *Restless*. In general, the dual system is more suitable for screening dominant mutants of *Geomyces* sp. WNF-15A. It indicated that reducing the carrier size appropriately would benefit the functioning of transposable elements.

In our previous study, transposable systems of *Impala*, *Fot1* and *Helitron* were constructed and applied in *Geomyces* sp. WNF-15A. Two normal temperature-adaptive mutants for red pigment production, i.e., MP2 (OD_520_ of 2.2) and MP10 (OD_520_ of 3.2), were obtained (Ding et al. 2021). However, the red pigment production of mutants adapted to normal temperature was higher in this study. The positive mutation ratios of *Minos* and *Restless* were much better than that of *Impala* (2.6%), *Fot1* (4.7%) and *Helitron* (9.4%) (Ding et al. 2021). Therefore, we proved the good activities of *Minos* and *Restless* transposons in polar fungus *Geomyces* sp. WNF-15A. However, the randomness of cut and insertion of *Minos* and *Restless* transposons in *Geomyces* sp. WNF-15A was still unclear and needs further studies. Although we successfully obtained normal temperature-adaptive mutants for red pigment synthesis, their production capacity was still weak in view of the industrial application. In the future, tracing of the transposon target and systematic rewiring of these genes may be performed to further improve red pigment production at normal temperature. Also, functions of the cold-adaptive genes are worth clarifying to provide more underlying mechanisms and direct the rational engineering of the normal temperature-adaptive strains for *Geomyces* sp. WNF-15A and other psychrotrophs.

## Conclusions

Available single and dual transposable systems of *Minos* and *Restless* transposons were developed and implemented in the polar fungus *Geomyces* sp. WNF-15A. Four mutants of normal temperature-adaptive red pigment synthesis and six mutants of normal-temperature growth-dominant were obtained and characterized. These mutants provide target samples for tracking cold-adaptive genes and will direct further rationalize rewiring of the *Geomyces* sp. WNF-15A. Our work also offers alternative tools for genetic mutagenesis breeding of other fungi living in cold habitats and producing functional products.

### Supplementary Information

**Additional file 1: Table S1.** Primers used in this study. **Figure S1.** Mutants screened by single transposable systems of *Minos* and *Restless*. **(a)** Cultured at 20℃. Right in each plate: wild-type (WT); Left in each plate: mutants (MPS1 ~ MPS10). MPS1 ~ MPS7, screened by *Minos* transposon; MPS8 ~ MPS10, screened by *Restless* transposon. **(b)** Cultured at 25 °C. WT, wild-type; MGS1 ~ MGS4, screened by *Minos* transposon; MGS5 ~ MGS6, screened by *Restless* transposon. * indicates a significant difference at *p* < 0.05, ** indicates a significant difference at *p* < 0.01. The pictures in figure were gained from different incubation time. The mutants were inoculated with the same number of spores as the wild-type. **Figure S2.** The red pigment production of mutants obtained by single transposable systems in fermentation. **(a)** Cultured at 14℃. **(b)** Cultured at 20℃. WT, wild-type; mutants MPS1 ~ MPS10, screened by single transposable systems; DW, biomass dry weight. Determination of absorbance value of red pigment at wavelength of 520 nm (OD_520_). **Figure S3.** The growth of mutants obtained by single transposable systems in fermentation at 25 °C. WT, wild-type; **(a)** MGS1. **(b)** MGS3. **(c)** MGS6, screened by single transposable systems; DW, biomass dry weight. **Figure S4.** The varying amplitude analysis of production and growth of positive mutants in liquid culture. The variation percentage in red pigment production of mutants cultured at 14 °C (a) and 20 °C (c) were shown. The variation percentage in cell growth of mutants cultured at 14 °C (b), 20 °C (d), and 25 °C (e) were shown. WT, wild-type; mutants MPS1 ~ MPS10, MGS1, MGS3 and MGS 6 screened by single transposable systems. **Figure S5.** Mutants screened by dual transposable systems of *Minos* and *Restless*. **(a)** Cultured at 20℃. Right in each plate, wild-type (WT); Left in each plate: mutants (MPD1 ~ MPD13). MPD1 ~ MPD6, screened by *Minos* transposon; MPD7 ~ MPD13, screened by *Restless* transposon. **(b)** Cultured at 25 °C. Mutants screened by dual transposable systems. WT, wild-type; MGD1 ~ MGD4, screened by *Minos* transposon; MGD5 ~ MGD8, screened by *Restless* transposon. * indicates a significant difference at *p* < 0.05, ** indicates a significant difference at *p* < 0.01. The pictures in figure were gained from different incubation time. The mutants were inoculated with the same number of spores as the wild-type. **Figure S6.** The red pigment production of mutants obtained by dual transposable systems in fermentation. **a (a)** Cultured at 14 °C. **(b)** Cultured at 20 °C. WT, wild-type; mutants MPD1 ~ MPD13, screened by dual transposable systems; DW, biomass dry weight. Determination of absorbance value of red pigment at wavelength of 520 nm (OD_520_). **Figure S7.** The growth of mutants obtained by dual transposable systems in fermentation at 25˚C. WT, wild-type; **(a)** MGD2. **(b)** MGD4. **(c)** MGD6, screened by dual transposable systems; DW, biomass dry weight. **Figure S8.** The varying amplitude analysis of production and growth of positive mutants in liquid culture. The variation percentage in red pigment production of mutants cultured at 14 °C **(a)** and 20 °C **(c)** were shown. The variation percentage in cell growth of mutants cultured at 14 °C **(b)**, 20 °C **(d)**, and 25 °C **(e)** were shown. WT, wild-type; mutants MPD1 ~ MPD13, MGD2, MGD4 and MGD6 screened by dual transposable systems.

## Data Availability

There is no data for submission in a public repository. Experimental materials and datasets for the current study are available from the corresponding author on reasonable request.
