# Correction: *Minos* and *Restless* transposon insertion mutagenesis of psychrotrophic fungus for red pigment synthesis adaptive to normal temperature

**DOI:** 10.1186/s40643-022-00622-3

**Published:** 2023-01-23

**Authors:** Fengning Lu, Yanna Ren, Lulu Ding, Jian Lu, Xiangshan Zhou, Haifeng Liu, Nengfei Wang, Menghao Cai

**Affiliations:** 1 State Key Laboratory of Bioreactor Engineering, East China University of Science and Technology, Shanghai, 200237 China; 2 China Resources Biopharmaceutical Co., Ltd, Unit 601, Building No. 2, YESUN Intelligent Community III, Guanlan Street, Shenzhen, China; 3 China Resources Angde Biotech Pharma Co., Ltd, 78 E-Jiao Street, Liaocheng, 252201 Shandong China; 4 First Institute of Oceanography, Ministry of Natural Resources, Qingdao, 266061 China; 5 Shanghai Frontiers Science Center of Optogenetic Techniques for Cell Metabolism, East China University of Science and Technology, 130 Meilong Road, Shanghai, 200237 China

**Correction: Bioresources and Bioprocessing (2022) 9:118** https://doi.org/10.1186/s40643-022-00604-5

In the original publication of the article, “Yanna Ren” should have been denoted as co-corresponding author. The original article (Fengning et al. 2022) has been corrected.
