# Supplementary material for: Minos and Restless transposon insertion mutagenesis of psychrotrophic fungus for red pigment synthesis adaptive to normal temperature
Source: Bioresour Bioprocess. 2022 Nov 4;9(1):118. doi: 10.1186/s40643-022-00604-5 (PMC10992017; doi:10.1186/s40643-022-00604-5)
Supplement: Supplementary file 1 — Additional file 1: Table S1. Primers used in this study. Figure S1. Mutants screened by single transposable systems of Minos and Restless. (a) Cultured at 20℃. Right in each plate: wild-type (WT); Left in each plate: mutants (MPS1 ~ MPS10). MPS1 ~ MPS7, screened by Minos transposon; MPS8 ~ MPS10, screened by Restless transposon. (b) Cultured at 25 °C. WT, wild-type; MGS1 ~ MGS4, screened by Minos transposon; MGS5 ~ MGS6, screened by Restless transposon. * indicates a significant difference at p < 0.05, ** indicates a significant difference at p < 0.01. The pictures in figure were gained from different incubation time. The mutants were inoculated with the same number of spores as the wild-type. Figure S2. The red pigment production of mutants obtained by single transposable systems in fermentation. (a) Cultured at 14℃. (b) Cultured at 20℃. WT, wild-type; mutants MPS1 ~ MPS10, screened by single transposable systems; DW, biomass dry weight. Determination of absorbance value of red pigment at wavelength of 520 nm (OD520). Figure S3. The growth of mutants obtained by single transposable systems in fermentation at 25 °C. WT, wild-type; (a) MGS1. (b) MGS3. (c) MGS6, screened by single transposable systems; DW, biomass dry weight. Figure S4. The varying amplitude analysis of production and growth of positive mutants in liquid culture. The variation percentage in red pigment production of mutants cultured at 14 °C (a) and 20 °C (c) were shown. The variation percentage in cell growth of mutants cultured at 14 °C (b), 20 °C (d), and 25 °C (e) were shown. WT, wild-type; mutants MPS1 ~ MPS10, MGS1, MGS3 and MGS 6 screened by single transposable systems. Figure S5. Mutants screened by dual transposable systems of Minos and Restless. (a) Cultured at 20℃. Right in each plate, wild-type (WT); Left in each plate: mutants (MPD1 ~ MPD13). MPD1 ~ MPD6, screened by Minos transposon; MPD7 ~ MPD13, screened by Restless transposon. (b) Cultured at 25 °C. Mutants screened by dual transposab [file 40643_2022_604_MOESM1_ESM.docx]

**Additional file 1**

***Minos* and *Restless* transposon insertion mutagenesis of psychrotrophic fungus for red pigment synthesis adaptive to normal temperature**

Fengning Lu^1^, Yanna Ren^1*^, Lulu Ding^1^, Jian Lu^1^, Xiangshan Zhou^2^, Haifeng Liu^3^, Nengfei Wang^4^, Menghao Cai^1,4*^

^1^ State Key Laboratory of Bioreactor Engineering, East China University of Science and Technology, Shanghai 200237, China

^2^ China Resources Biopharmaceutical Co., Ltd, Unit 601, Building No.2, YESUN Intelligent Community III, Guanlan Street, Shenzhen

^3^ China Resources Angde Biotech Pharma Co., Ltd., 78 E-jiao Street, Liaocheng, Shandong 252201, China

^4^ First Institute of Oceanography, Ministry of Natural Resources, Qingdao 266061, China

^5^ Shanghai Frontiers Science Center of Optogenetic Techniques for Cell Metabolism, East China University of Science and Technology, 130 Meilong Road, Shanghai 200237, China

Correspondence should be addressed to: Menghao Cai. E-mail: [cmh022199@ecust.edu.cn](mailto:cmh022199@ecust.edu.cn); Tel./fax: 86-21-64253286.

**Table S1 Primers used in this study.**

| **Primers** | **Sequence (5’-3’)** |
| --- | --- |
| HM-F1 | AGAAGAGCTCAGTACCCATGAAGCATCCAAATGCCTGAAC |
| HM-R1 | TTCGAACGATATCACCATGGCTCTATTCCTTTGCCCTCGG |
| HR-F1 | ATCCAATCTCTCAGTACAGGATGCCTGAACTCACCGCGAC |
| HR-R1 | CTACTCTGAGCTCTTCTAGGCTATTCCTTTGCCCTCGGACGAG |
| HM-DF1 | AACACAACCTTCACCTTAATGACACTTACGGAGATAAGCA |
| HM-DR1 | TTGAATCGCGCATTGGATCCAAGGGTTTTTCCTTGGCTTT |
| HM-HF1 | TCGAACGCGGCGTAAACCGGCGTTTGACAAGATGGTTCAT |
| HM-HR1 | TTAAGCTCGGGCCCCCTGGGTTCTAGATTGGATGCTTGGG |
| HR-DF1 | AACACAACCTTCACCTTAATGCACTCAGACTCTGACAGAAC |
| HR-DR1 | AATCGAATGTCCGCGCATGCGATAGATCGCAGTCGAGATCG |
| HR-HF1 | GCGTAATCAACCAAACCGGTCCAGGCGGATCACAAAATTT |
| HR-HR1 | TTAAGCTCGGGCCCCCTGGGTCGGCATCTACTCTGAGCTC |
| Am-F | GGTACCAATCTGCAGTATGCTTGATG |
| Am-R | CAAGAAAATGATGCTCGGAATCCAT |
| Mi-F | CGTTTGTCAAGCAAGGTAAGTGA |
| Mi-R | CGCTGAGGTCTTAATGCGGT |
| Re-F | CGCCCTTCCTCCCTTTATTTCAGA |
| Re-R | CGCTGAGGTCTTAATGCGGT |
| MD-F | GACTTACCTATTCTACCCAAGCAT |
| MD-R | CCACTATTAATTCGAACCTCGTGG |
| RD-F | AGCAGACAGGAACGAGGACA |
| RD-F | ACACAGCCATCGGTCCAGAC |


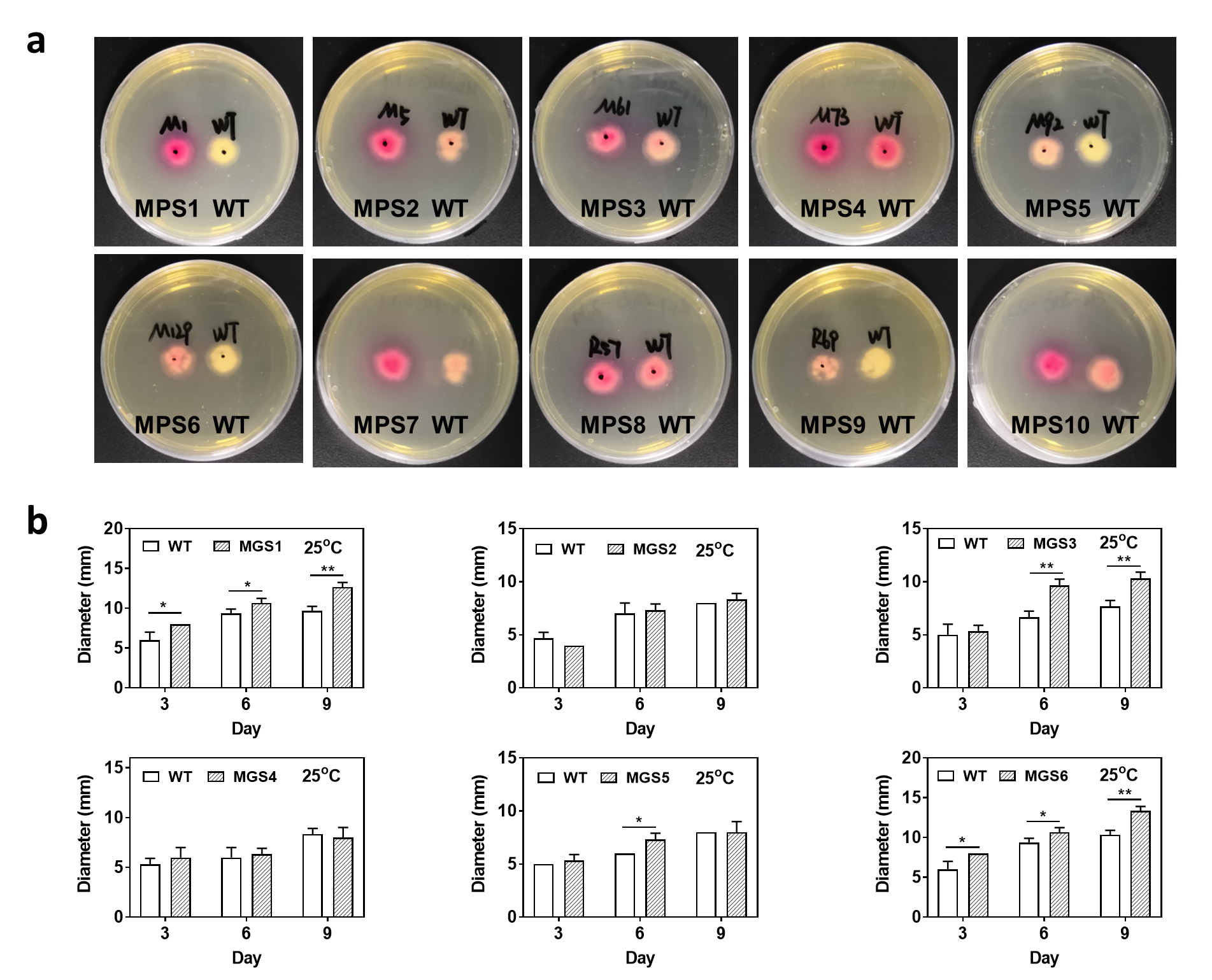


**Fig. S1** Mutants screened by single transposable systems of *Minos* and *Restless*. **(a)** Cultured at 20℃. Right in each plate: wild-type (WT); Left in each plate: mutants (MPS1~MPS10). MPS1~MPS7, screened by *Minos* transposon; MPS8~MPS10, screened by *Restless* transposon. **(b)** Cultured at 25°C. WT, wild-type; MGS1~MGS4, screened by *Minos* transposon; MGS5~MGS6, screened by *Restless* transposon. * indicates a significant difference at *p*<0.05, ** indicates a significant difference at *p*<0.01. The pictures in figure were gained from different incubation time. The mutants were inoculated with the same number of spores as the wild-type.


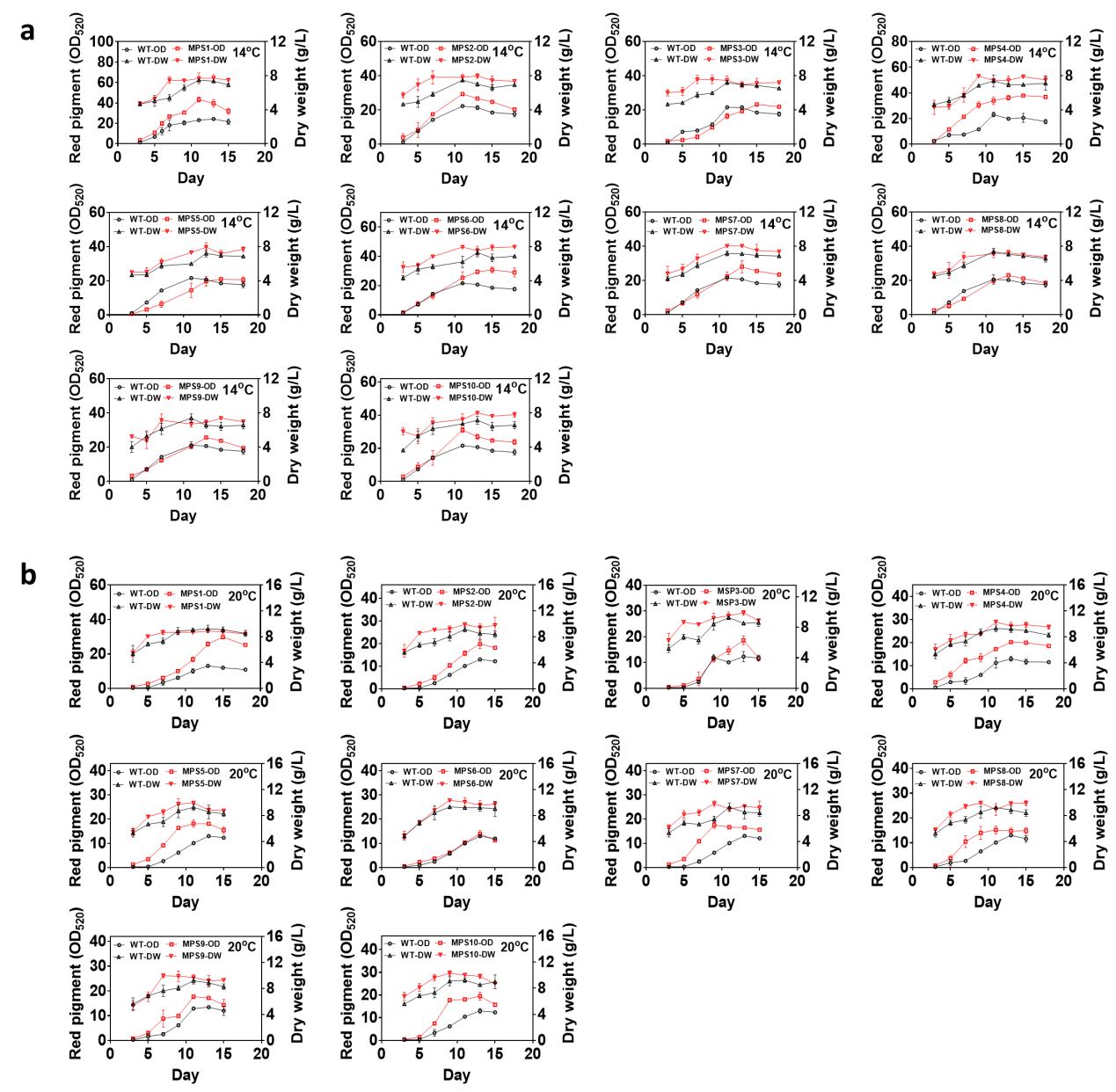


**Fig. S2** The red pigment production of mutants obtained by single transposable systems in fermentation. **(a)** Cultured at 14℃. **(b)** Cultured at 20℃. WT, wild-type; mutants MPS1~MPS10, screened by single transposable systems; DW, biomass dry weight. Determination of absorbance value of red pigment at wavelength of 520 nm (OD_520_).


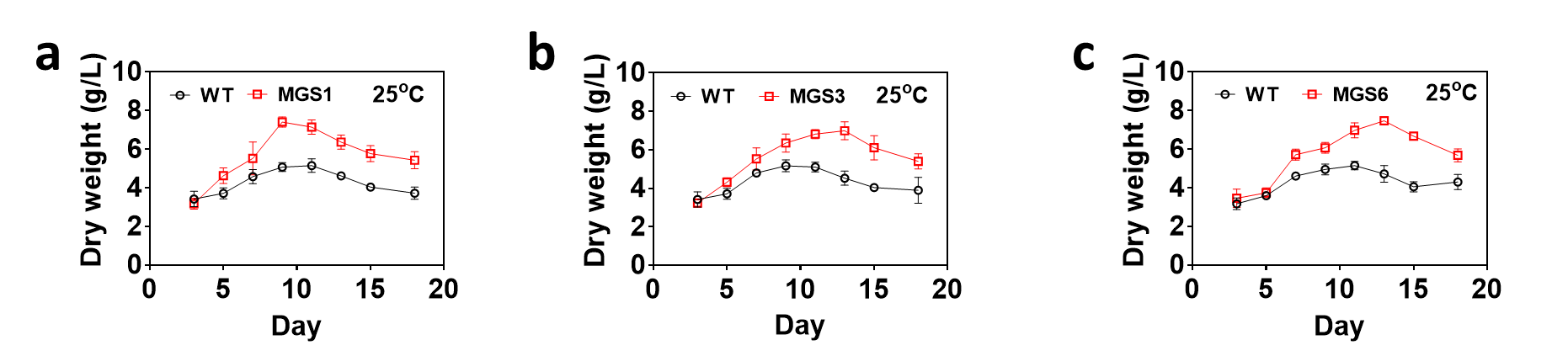


**Fig. S3** The growth of mutants obtained by single transposable systems in fermentation at 25°C. WT, wild-type; **(a)** MGS1. **(b)** MGS3. **(c)** MGS6, screened by single transposable systems; DW, biomass dry weight.


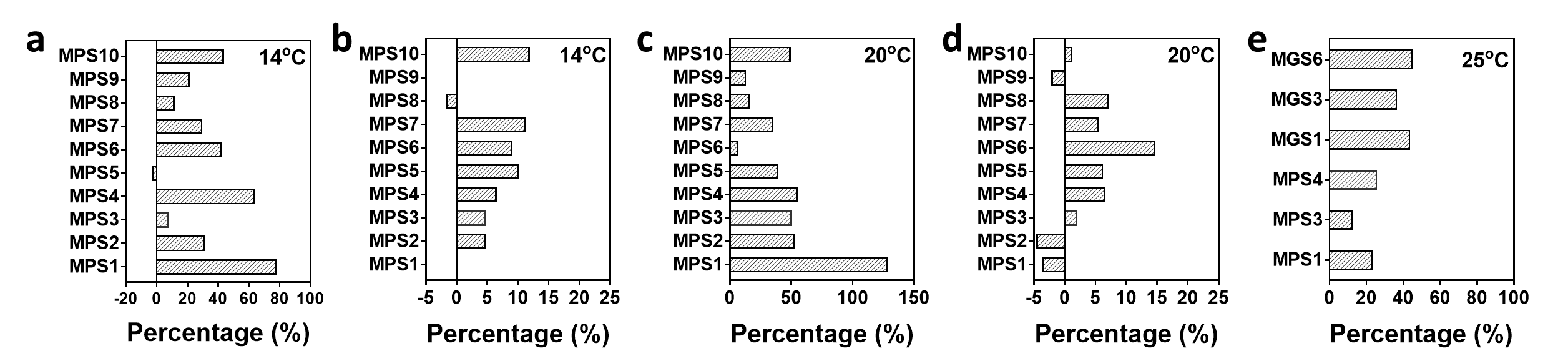


**Fig. S4** The varying amplitude analysis of production and growth of positive mutants in liquid culture. The variation percentage in red pigment production of mutants cultured at 14°C (a) and 20°C (c) were shown. The variation percentage in cell growth of mutants cultured at 14°C (b), 20°C (d), and 25°C (e) were shown. WT, wild-type; mutants MPS1~MPS10, MGS1, MGS3 and MGS 6 screened by single transposable systems.


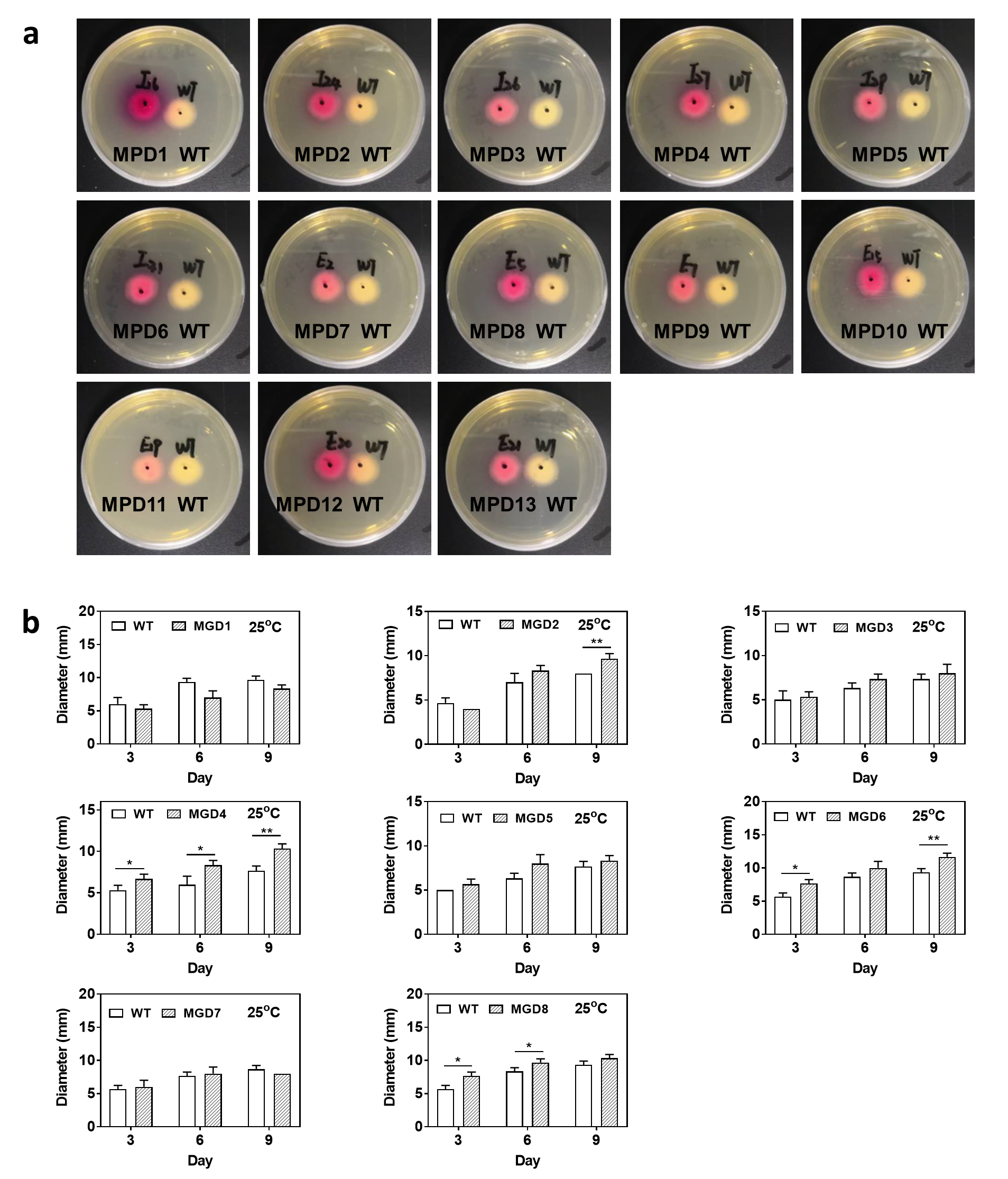


**Fig. S5** Mutants screened by dual transposable systems of *Minos* and *Restless*. **(a)** Cultured at 20℃. Right in each plate, wild-type (WT); Left in each plate: mutants (MPD1~MPD13). MPD1~MPD6, screened by *Minos* transposon; MPD7~MPD13, screened by *Restless* transposon. **(b)** Cultured at 25°C. Mutants screened by dual transposable systems. WT, wild-type; MGD1~MGD4, screened by *Minos* transposon; MGD5~MGD8, screened by *Restless* transposon. * indicates a significant difference at *p*<0.05, ** indicates a significant difference at *p*<0.01. The pictures in figure were gained from different incubation time. The mutants were inoculated with the same number of spores as the wild-type.


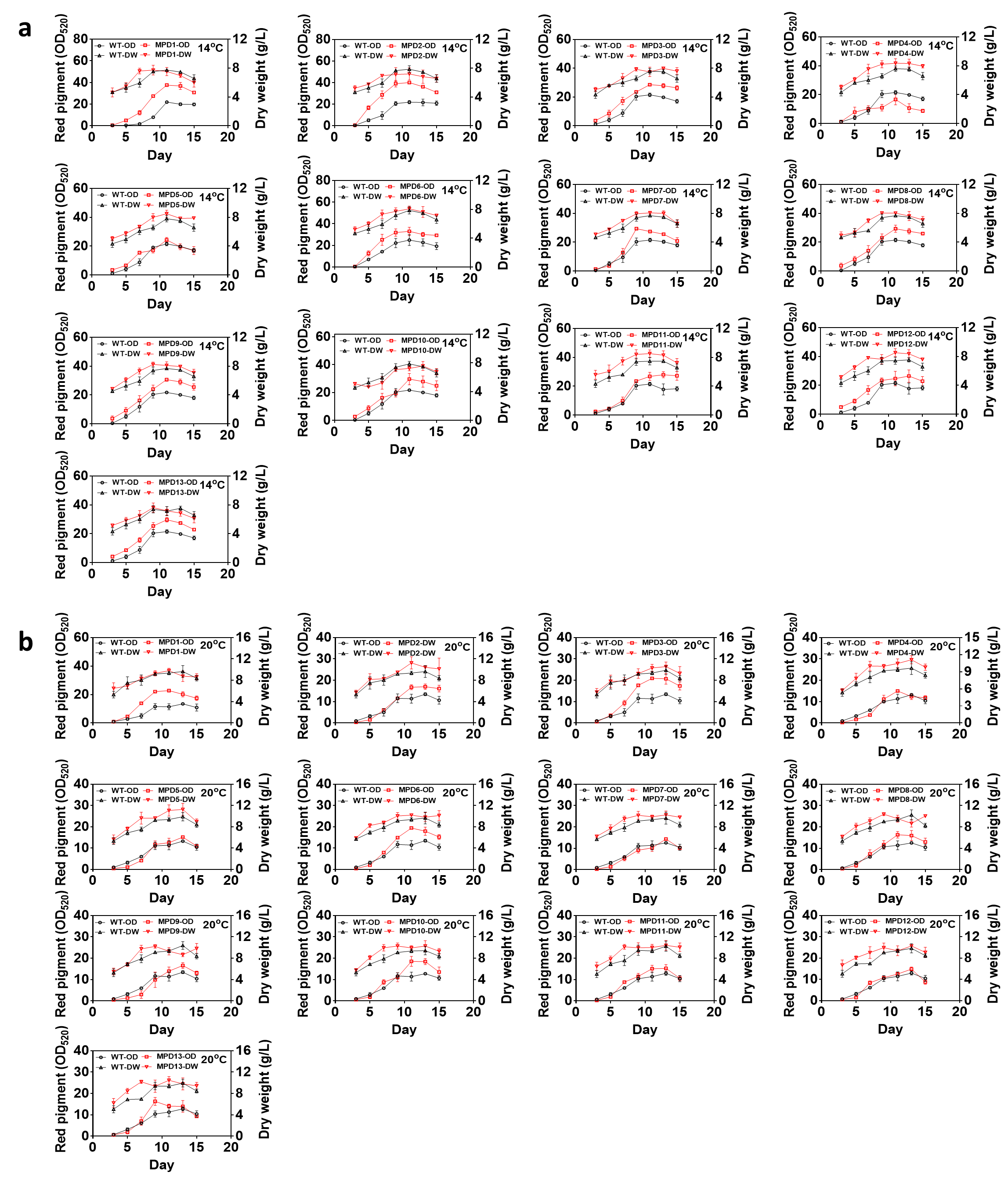


**Fig. S6** The red pigment production of mutants obtained by dual transposable systems in fermentation. **a (a)** Cultured at 14°C. **(b)** Cultured at 20°C. WT, wild-type; mutants MPD1~MPD13, screened by dual transposable systems; DW, biomass dry weight. Determination of absorbance value of red pigment at wavelength of 520 nm (OD_520_).


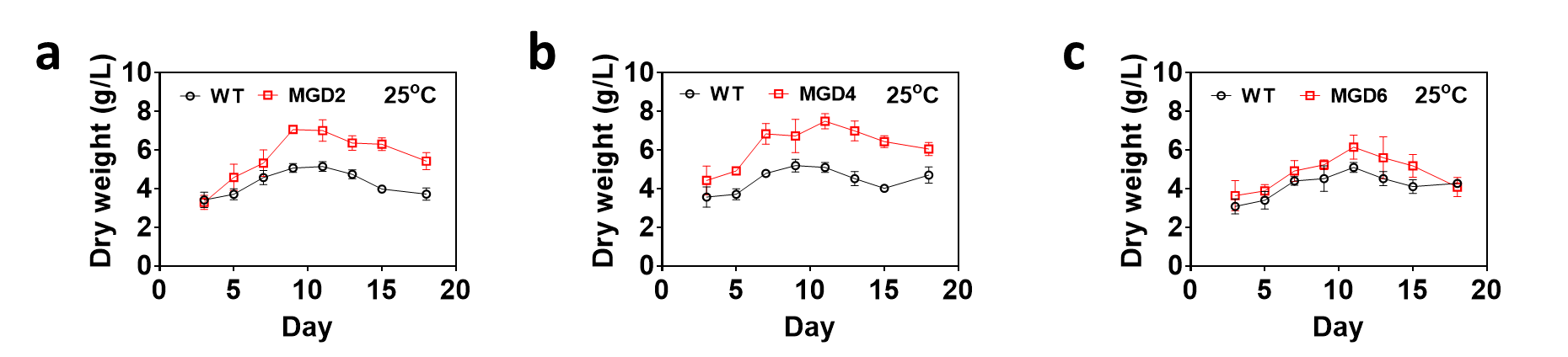


**Fig. S7** The growth of mutants obtained by dual transposable systems in fermentation at 25˚C. WT, wild-type; **(a)** MGD2. **(b)** MGD4. **(c)** MGD6, screened by dual transposable systems; DW, biomass dry weight.


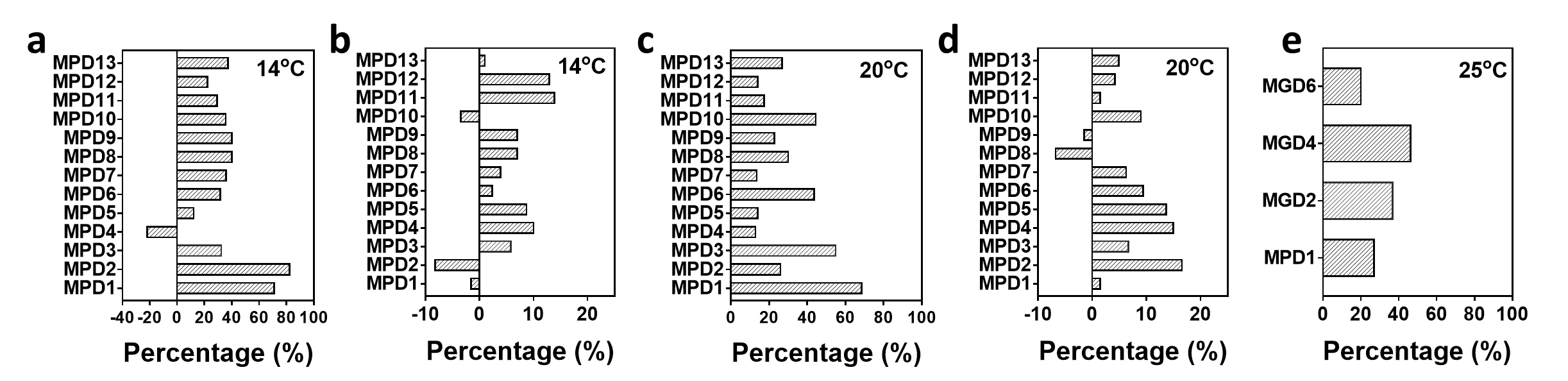


**Fig. S8** The varying amplitude analysis of production and growth of positive mutants in liquid culture. The variation percentage in red pigment production of mutants cultured at 14°C **(a)** and 20°C **(c)** were shown. The variation percentage in cell growth of mutants cultured at 14°C **(b)**, 20°C **(d)**, and 25°C **(e)** were shown. WT, wild-type; mutants MPD1~MPD13, MGD2, MGD4 and MGD6 screened by dual transposable systems.
